# Activation of Extraoral Bitter Taste Receptors by Herbs and Spices in the Regulation of Incretin Signaling and Glucose Metabolism

**DOI:** 10.1093/nutrit/nuag031

**Published:** 2026-05-26

**Authors:** Slavko Komarnytsky, Reham Mhawish, Charles Wagner

**Affiliations:** Plants for Human Health Institute, North Carolina State University, Kannapolis, NC, 28081, United States; Department of Food, Bioprocessing, and Nutrition Sciences, North Carolina State University, Raleigh, NC, 27695, United States; Plants for Human Health Institute, North Carolina State University, Kannapolis, NC, 28081, United States; Department of Food, Bioprocessing, and Nutrition Sciences, North Carolina State University, Raleigh, NC, 27695, United States; Department of Nutrition and Food Technology, Jordan University of Science and Technology, Irbid, 22110, Jordan; Plants for Human Health Institute, North Carolina State University, Kannapolis, NC, 28081, United States

**Keywords:** spices, culinary herbs, bitters, bitter receptors, extraoral, gastrointestinal modulation, glucose metabolism, postprandial glucose, blood sugar spikes, hypoglycemic

## Abstract

Bitter compounds in plants, originally evolved as chemical defenses, are retained within the dry matrices of spices and culinary herbs, where they constitute a chemically diverse pool of potential ligands for bitter taste receptors (TAS2Rs) expressed along the gastrointestinal tract. Beyond their role in oral taste perception, TAS2Rs are increasingly recognized as extraoral chemosensory receptors capable of modulating intestinal physiology. Evidence suggests that activation of specific TAS2Rs by bitter phytochemicals can influence glucose transporter activity and stimulate the release of enteroendocrine hormones, including glucagon-like peptide-1 and cholecystokinin, thereby engaging established pathways involved in appetite regulation and glucose homeostasis. These shared downstream effects may explain why structurally diverse bitter phytochemicals often produce convergent metabolic outcomes. Framing bitter-receptor signaling within known enteroendocrine mechanisms highlights an underexplored opportunity to leverage herbs and spices as dietary modulators of metabolic function. Incorporating naturally bitter plant foods into dietary patterns may offer a simple, sustainable, and culturally adaptable approach to support metabolic health.

## INTRODUCTION

Humans recognize bitter substances through a diverse group of about 25 functional bitter taste receptors (TAS2Rs).^1^ They appeared for the first time in cartilaginous and bony fishes around 430-460 million years ago and rapidly expanded in vertebrates during the subsequent transition from aquatic to terrestrial life.^2^^,^^3^ This expansion coincided with the emergence of bitter plants and insects that became major dietary sources for the early vertebrates.^4^ Animal prey generally lack bitter-tasting substances, with a primary exception of bile^5^; thus, the development of a broad and sensitive bitter detection system conferred a strong survival advantage by enabling navigation and feeding in an increasingly complex terrestrial chemical environment—for example, by detection or avoidance of bitter plant alkaloids,^6^ glucosinolates,^7^ or phenolic glycosides.^8^

Calling TAS2Rs “bitter receptors,” however, was a significant disservice to their true biological chemosensory role and vast extraoral functionality, similar to other taste-receptor families.^9^ The expansion and diversification of bitter taste receptors in the gut, airways, and immune cells led to their evolutionary repurposing for additional metabolic and immune functions.^10^ Today, there is growing support for the notion that bitter substances routinely engage chemosensory receptors all over the human body. In the gut, these receptors are found on enteroendocrine cells, epithelial cells, and some immune cell populations, where they can sense dietary and microbial-derived bitter compounds.^11^ Activation of gut TAS2Rs triggers intracellular signaling cascades that can stimulate the secretion of hormones that regulate glucose metabolism, appetite, and gut motility.^12^ These receptors also modulate local defense mechanisms, including antimicrobial peptide release and barrier function, linking chemosensation to gut immune responses.^13^ Importantly, the sensitivity and responsiveness of TAS2Rs can vary between individuals, reflecting both genetic variation in receptor expression and adaptation to habitual diet.^14^ A well-characterized example is the perception differences of 6-*n*-propylthiouracil and phenylthiocarbamide that arise from common polymorphic variants in TAS2R38.^15^ However, extraoral gastrointestinal TAS2Rs function independently of oral bitterness perception, indicating that individuals with differing oral sensitivity can still exhibit robust gut TAS2R activation.

With terrestrial adaptation, plants also became the dominant dietary sources of carbohydrates, supplying starches, sugars, and fermentable fibers that supported expanding human metabolic demands. Consequently, hominids were faced with the dual challenge of extracting energy from plant-derived carbohydrates while simultaneously ingesting the bitter phytochemicals that frequently co-occur in the same tissues.^16^ This evolutionary pressure likely favored the coupling of bitter chemosensing with metabolic control systems that regulate intestinal glucose handling and postprandial hormone release.^17^ This makes TAS2Rs especially relevant for engineering foods that deliver metabolic signals without adding digestible energy, distinguishing them conceptually from TAS1R-driven pathways. The focus on TAS2Rs in this review does not imply that bitter tastants uniquely regulate gut hormone secretion; rather they represent a distinct and underutilized chemosensory entry point for modulating shared metabolic pathways.

Spices and herbs are uniquely positioned to leverage this system, offering not just flavor but also potential modulatory effects through their concentrated bitter-tasting constituents.^18^ The structural diversity of bitter compounds in herbs and spices allows for engagement of multiple TAS2Rs,^19^ providing a broad signaling repertoire that can be tailored to individual sensitivities and dietary patterns. By incorporating bitter spices and herbs into the diet, it may be possible to harness these evolutionary chemosensory pathways to support metabolic health and modulate postprandial energy handling. However, a significant knowledge gap remains in understanding how these compounds interact with TAS2Rs at the molecular level across different tissues and how they can be effectively used for health-promoting purposes. In this narrative review, we address this gap by focusing on the effects of bitter-tasting substances on carbohydrate metabolism and, specifically, their influence on glucose uptake and appearance in the blood, using both human clinical data and cell culture models.

## METHODS

This narrative review was developed through a summary of our work on the subject combined with an extensive search of scientific literature using databases including PubMed, Google Scholar, Web of Science, and Scopus accessed via institutional subscriptions at North Carolina State University, covering publications up to December 2025. Only peer-reviewed studies published in English were considered. The review concentrated on research published in the past 10 years. Keywords used in the search strategy included the following: “bitter taste receptors” OR “TAS2Rs” OR “bitter compounds” OR “phytochemicals” AND “glucose regulation” OR “GLP-1” OR “CCK” OR “postprandial glucose” OR “glucose absorption” OR “glucose metabolism” AND “spices” OR “herbs” OR “bitters” OR “culinary bitterness” OR “dietary interventions” OR “metabolic health.”

## SPICES AND HERBS IN TRADITIONAL BITTER PREPARATIONS

The use of spices and herbs predates modern humans, as evident from dental calculus of Neanderthals from El Sidrón cave in northern Spain dated to 50 600-47 300 BCE that contained bitter-tasting dihydroazulene and chamazulene found in yarrow (*Achillea millefolium* L.) and chamomile (*Matricaria chamomilla* L.).^20^ The same individuals were also heterozygous tasters, because divergent alleles of TAS2R38 were maintained.^21^ This, together with a substantial expansion of α-amylase *AMY1* copy variants in the genomes of early hominids, points to a prominent dietary transition toward increased consumption of plants and storage carbohydrates (tubers, roots, nuts, and grains) outside of rainforest areas.^22^ Because many wild plant foods naturally harbor bitter phytochemicals, the increase of carbohydrate loads in human diets also increased the consumption of plants containing bitter phytochemicals. On the other hand, additional exposure of foods to fermentation or heating (Maillard reactions) also generates bitter-tasting substances such as hydrophobic peptides, quinones, furanones, and pyrazines.^23^ Partial debittering of plant foods was achieved in the form of cooking^24^^,^^25^ or nixtamalization with hot limestones,^26^ but it was not until the development of modern crop cultivars and industrial debittering that human diets experienced significant reduction of bitter tastes.^27^ Consistent with this knowledge, modern higher-quality diets are intrinsically more bitter, and promoting greater acceptance of bitter flavors could contribute to improved dietary patterns in the general population.^28^

Inherent bitterness of spices and herbs found its way into many formulations dating back to classical antiquity.^29^ Bitter-tasting mithridate and theriaca (also called tiryaq and treacle) remedies in the form of honey electuary typically contained gentian, St. John’s wort, parsley, anise, ginger, and cinnamon in a complex mixture of up to 70 ingredients, as recorded by Celsus and Galen, and targeted the gastrointestinal tract as an antidote to ingested poisons.^30^^,^^31^ The importance of consuming bitter herbs was also recognized in many traditional texts (eg, as a choice of *maror* [Hebrew, meaning “with bitter herbs they shall eat it”]). This tradition continued in the form of bitter aperitifs or digestifs targeting gastrointestinal health, such as Chartreuse and Bénèdictine (France); amaro, Fernet, and Campari (Italy); Kräuterlikör, Jägermeister, and Underberg (Germany); Appenzeller Alpenbitter (Switzerland); Becherovka (Czechoslovakia); and Angostura and Peychaud’s (Americas), among others. Although their bitter profiles vary, they are typically dominated by gentian, wormwood, angelica, hyssop, anise, caraway, yarrow, bitter orange, and cinchona.^32^ Modern research supports the traditional use of these preparations, showing that bitter mixtures can stimulate digestive secretions, modulate gut motility, and affect satiety, as we discuss later in this review.

## DIVERSITY OF BITTER PHYTOCHEMICALS IN SPICES AND HERBS

Spices are defined by the US Food and Drug Administration as any “aromatic vegetable substances … whose significant function in food is seasoning”^32^. The term “herbs” is traditionally restricted only to dry aerial parts (leaves and flowers). In modern times, consumption of spices varies from 0.5 g d^–1^ in Europe to 1.8 g d^–1^ in Africa and 2.6-4.4 g d^–1^ in Asia and Latin America for an average adult.^33^ Although spices and herbs are traditionally used for their modulation of taste, flavor, color, texture, or food preservation, their bitter compounds contribute not only to the sensory complexity of culinary ingredients but also to their functional roles as chemical defenses in plants.^34^ It is plausible, therefore, that many bitter phytochemicals evolved to interact with conserved chemosensory pathways in the mammalian gastrointestinal system, suggesting a co-evolutionary chemical dialogue between plants and animal physiology, particularly in the upper gastrointestinal tract, much like the well-established bitter-signaling crosstalk between the microbiome and the colon.^35^

Bitterness is a common sensory property of many spices and herbs. Table 1 presents a brief and incomplete summary of the distribution of bitter compounds across botanicals, their phytochemical classifications, and known activations of human bitter-taste receptors.^36^^,^^37^ The data set highlights a broad distribution of bitter phytochemicals across different plant tissues and chemical classes. Alkaloids are the most common bitter compounds, appearing in barks (quinine), fruits (berberine, piperine), seeds (xylopine, theobromine), and leaves (skimmianine), and activating a wide range of the TAS2Rs. Sesquiterpene lactones, notably in flowers (eg, chamomile), leaves (eg, wormwood), and roots (eg, dandelion, chicory), show strong activation of TAS2R46, suggesting this receptor plays a central role in detecting plant-derived lactones. Some receptors, such as TAS2R14 and TAS2R46, are frequently activated by multiple compound classes, indicating broad ligand specificity. In contrast, receptors like TAS2R2 and TAS2R16 are selectively activated by specific compounds (eg, curcumin and sinigrin, respectively), suggesting narrow functional roles. Certain phytochemicals, like amarogentin and humulone, are effective at very low concentrations, reflecting high receptor sensitivity (Table 1).

**Table 1.** Bitter Principles in Common Spices and Herbs, and the Corresponding Bitter-Receptor Activation Profiles, Summarized After Bayer et al^36^ and Zaikin et al^37^

Plant tissue	Spice or herb	Bitter principle	Phytochemical group	**TAS2R** ^a^ **activation (effective concentration, µM)**
Bark	Cinchona	Quinine	Alkaloids	TAS2R1, 4, 7, 10, 14, 39, 40, 41, 43, 44, 46 (10-1000)
	Cinnamon	Coumarin	Coumarins	TAS2R10,14 (300)
	Quassia	Quassin	Triterpene lactones	TAS2R4, 10, 14, 30, 46, 47 (300)
Flowers	Chamomile	Nobilin	Sesquiterpene lactones	TAS2R46 (0.1)
	Clove	Gallic acid	Gallotannins	TAS2R4, 14 (0.2-220)
	Hops	Humulone	α Acids	TAS2R1, 14, 40, 47 (0.01-30)
	Saffron	Picrocrocin	Monoterpene glycosides	Unknown (22)
Fruits	Barberry	Berberine	Alkaloids	TAS2R38, 46 (10)
	Bitter orange (also called chenpi)	Naringin	Flavanone glycosides	Unknown (10-220)
	Pepper, black	Piperine	Alkaloids	TAS2R14 (10)
Leaves	Basil, oregano	Rosmarinic acid	Caffeic acid esters	Unknown (103)
	Parsley	Apigenin	Flavone glycosides	TAS2R14, 39, 43 (1-30)
	Rosemary, sage	Carnosic acid	Diterpenes	Unknown
	Rue (ruta)	Skimmianine	Alkaloids	TAS2R14
	Wormwood	Absinthin	Sesquiterpene lactones	TAS2R10, 14, 46, 47 (0.1-100)
Roots	Angelica	Furanocoumarins	Furanocoumarins	TAS2R10, 14, 49
Rhizomes	Chicory	Lactucopicrin	Sesquiterpene lactones	TAS2R43, 46
	Dandelion	Taraxacin	Sesquiterpene lactones	TAS2R46 (0.1-100)
	Gentian	Amarogentin	Secoiridoid glycosides	TAS2R1, 4, 39, 43, 46, 47, 50 (3-300)
	Turmeric	Curcumin	Curcuminoid	TAS2R2
Seeds	Cacao	Theobromine	Alkaloids	TAS2R14 (1000)
	Celery	Butylphthalide	Phthalide lactones	Unknown
	Fenugreek	Diosgenin	Saponins	Unknown
	Grains of Selim (also called diarr)	Xylopine	Alkaloids	Unknown
	Hyssop	Marrubiin	Diterpene lactone	TAS2R46 (0.3)
	Mustard	Sinigrin	Glucosinolates	TAS2R16, 38 (100)
	Nigella (also called qizha)	Thymoquinone	Quinones	Unknown

a TAS2R, family of bitter-taste receptor compounds.

## BITTER RECEPTORS FROM A FUNCTIONAL PERSPECTIVE

**Figure 1.**
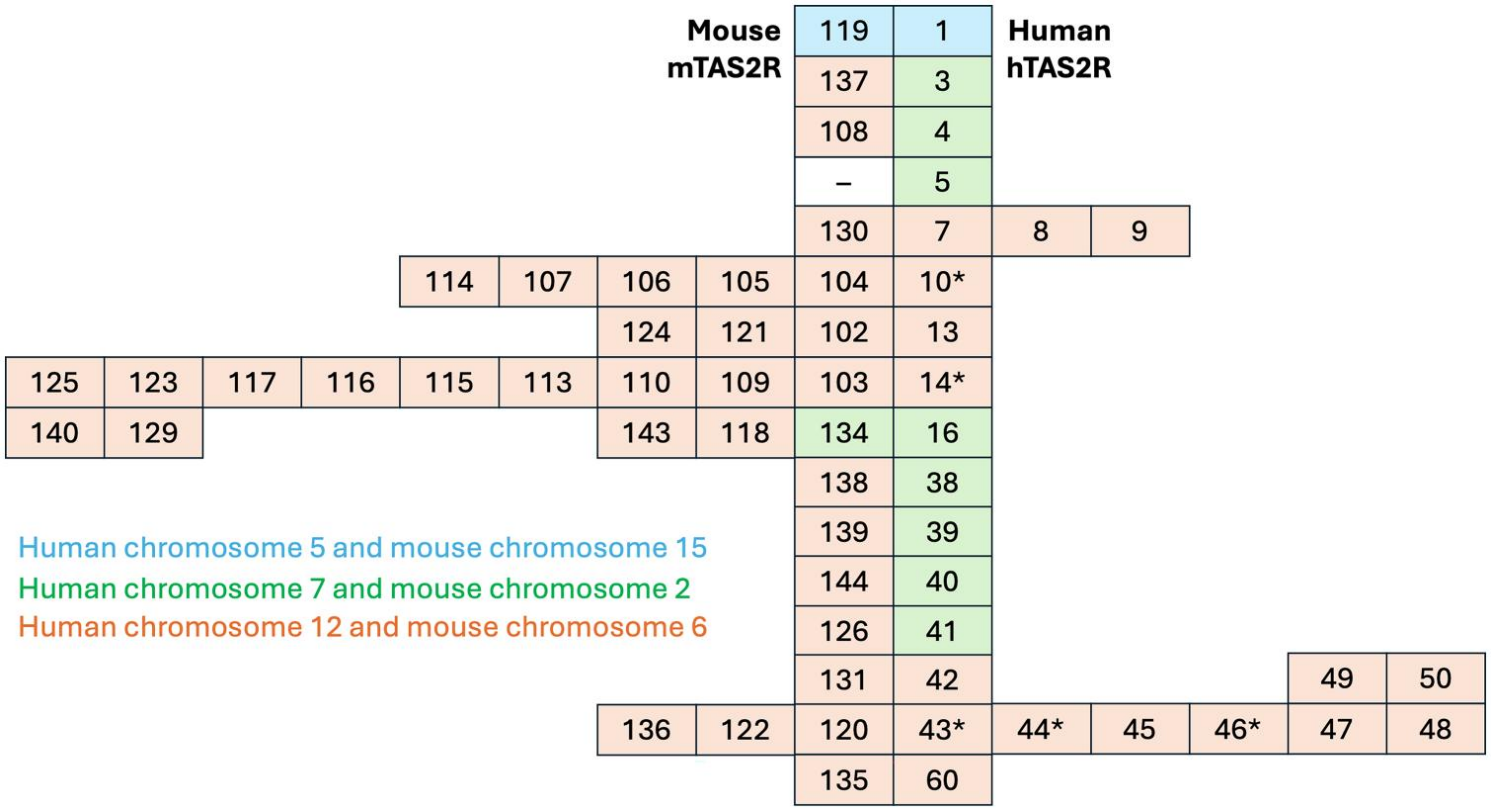
Evolutionary relationships between the ortholog human (hTAS2R) and mouse (mTAS2R) bitter-taste receptors, summarized after Hayakawa et al^40^ and Lossow et al.^41^ Clustering is based on multiple sequence alignment of the individual bitter receptors; their chromosomal localization is color coded. Human TAS2R2, 12 (26), 15, 18, 62, 63, and 64 are not listed, due to nonfunctional pseudogene status. Human TAS2R44 (31), 47 (30), 48 (19, 23), 49 (20), and 50 (51) gene names are synonymous. Human TAS2R5 does not seem to have an ortholog in mice. *Human TAS2Rs with broad specificity.

Classical TAS2Rs in the oral cavity enable a general aversion to the unpleasant bitter taste as an early signal to avoid ingestion of poisonous plants, insects, scavenged animal carcasses, and other spoiled foods.^38^ In primates, the number of functional TAS2R genes varies from 18 to 26, whereas humans maintain 25 active TAS2R genes and 8 nonfunctional pseudogenes, all clustered on 3 chromosomes.^39^ This clustering is evolutionarily preserved in mammals: mouse mTAS2R genes exist in 3 similar clusters, although some subgroups of the TAS2R genes show a clear tendency for both expansion and contraction.^40^^,^^41^ This process may have increased or decreased functional redundancy of bitter-taste perception, as well as allowed for additional new functionality of the broad-specificity human TAS2R10, TAS2R14, TAS2R43, and TAS2R46 genes that also exist as the expanded mTAS2R gene subgroups in mice (Figure 1). Expansion of TAS2R gene clusters had a clear evolutionary advantage on land: their number peaked at 74 loci in coelacanths, 50-136 loci in anuran frogs, and 36-50 loci in lizards.^42^ Yet there was no advantage of TAS2R functionality in the marine environment: birds (penguins) and mammals (cetaceans) that returned to the ocean experienced a near-complete loss of TAS2Rs.^43^^,^^44^

The second notable feature of the TAS2Rs is their spatial distribution. Beyond the classical localization throughout the oral cavity and the increased abundance in its posterior part, where TAS2Rs recognize bitter gustatory stimuli and lingering bitter aftertastes,^45^^,^^46^ these receptors can be also found in other tissues exposed to the external environment, such as the respiratory, urinary, and extraoral gastrointestinal systems, where they operate independently of conscious taste. In those locations, they are often associated with the ciliated epithelial cells and contribute to innate immune defenses, production of type 2 immune cytokines IL-4 and IL-13, and prevention of pathogen invasion.^47^^,^^48^ Additionally, bitter ligands induce relaxation of smooth muscles in airways,^49^ the vascular system,^50^ and the gut, where the gastric emptying is also delayed.^51^ Many of the blood cells express functional TAS2Rs, including leukocytes^52^ and monocytes^53^ that seem to respond to bitter ligands with the chemotactic transmigration. Because blood cells, as well as brain and heart tissues, are not directly exposed to the external environment, there is also a high chance that endogenous TAS2R ligands exist, as has been shown for bile acids (namely, TAS2R1, TAS2R4, TAS2R14, TAS2R39, TAS2R46)^54^ and bitter peptides.^55^

### Gastrointestinal Bitter Receptors and Neuroendocrine Regulation

**Figure 2.**
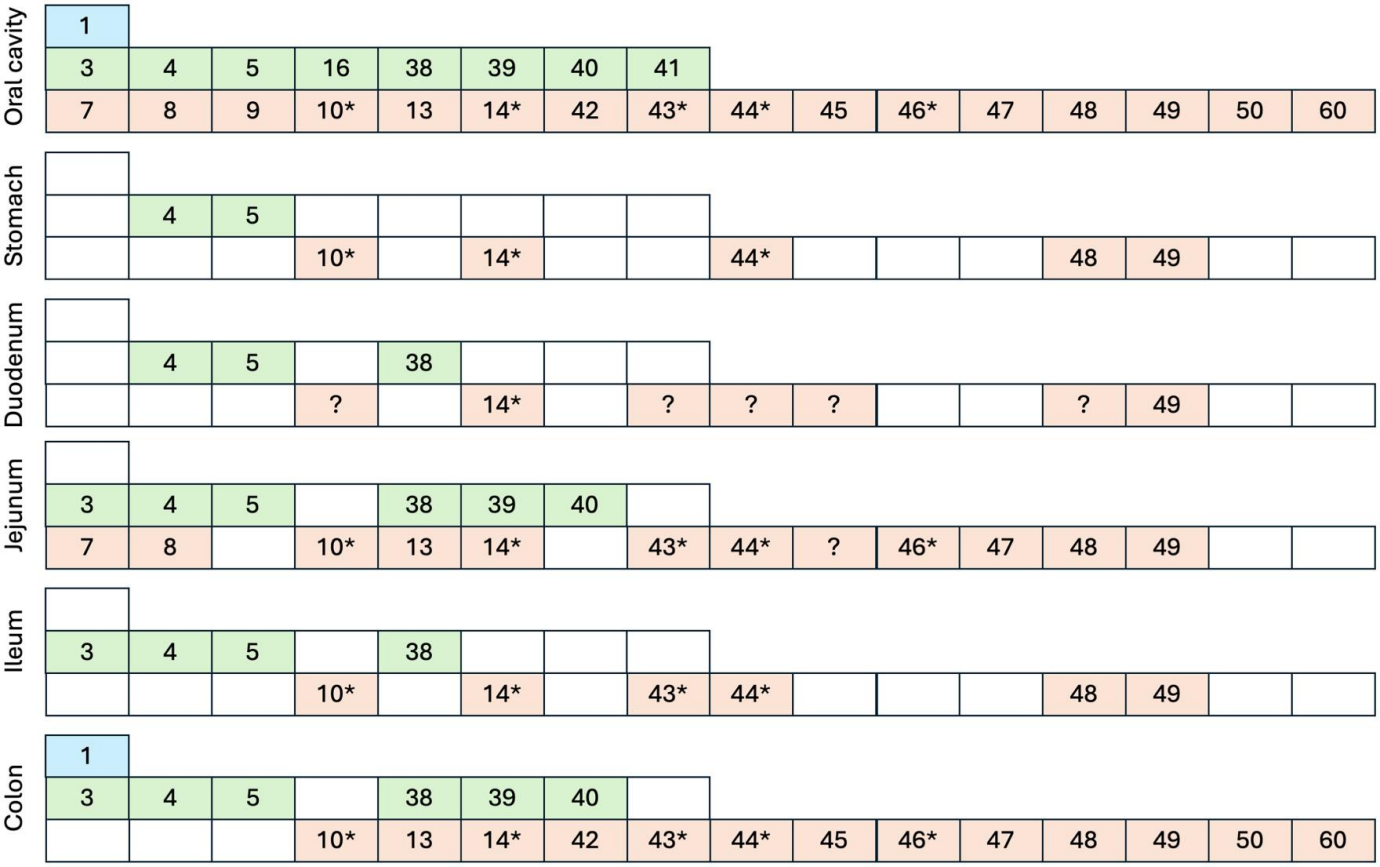
Expression profile of the bitter taste receptors (TAS2Rs) in the different regions of the human gastrointestinal tract, summarized after Descamps-Solà et al.^58^ Human TAS2R44 (31), 47 (30), 48 (19, 23), 49 (20), and 50 (51) gene names are synonymous.*Human TAS2R with broad specificity. ?, no current data availability.

The extraoral distribution of the human TAS2Rs follows several clear trends. Whereas all 25 TAS2Rs are expressed in the oral cavity, the colon tissues do not express a cluster of the related receptors TAS2R7, TAS2R8, and TAS2R9 (mouse ortholog mTAS2R130), as well as 2 receptors with broad specificity: TAS2R16 and TAS2R41 (mouse ubiquitous orthologs mTAS2R143 and mTAS2R126).^56^ This abundance of bitter receptors may be driven by higher amounts of microbiota and the microbial bitter ligands at these sites. Finally, 2 TAS2R genes from chromosome 7—TAS2R4 and TAS2R38—are ubiquitously expressed throughout the gut, and this pattern is evolutionally conserved (mouse orthologs mTAS2R108 and mTAS2R138).^57^ The ubiquitous gut expression of related TAS2R48 and TAS2R49 is also observed in humans, but not rodents that express mTAS2R143 and mTAS2R126 instead^58^ (Figure 2).

The early connection between TAS2Rs, bitter chemosensing, and metabolic regulation was established when a large number of the TAS2R promoters were reported to contain the binding sites for SREBP-2, indicating that dietary cholesterol levels may modulate intestinal TAS2R expression, although the precise signaling cascade remains to be fully established.^59^ TAS2R stimulation of cholecystokinin (CCK) secretion was also enhanced directly by SREBP-2 in cultured cells and in mice.^59^ These findings were also extended to glucagon-like peptide-1(GLP-1) in the Amish Family Diabetes Study.^60^ The effects on TAS2R gene expression and correlation with GLP-1 increases in response to different classes of bitter plant phytochemicals were confirmed in a preclinical model.^61^ The colocalization of TAS2R5 and GLP-1 was also confirmed in human duodenal and ileac tissues.^62^ It is likely, therefore, that bitter phytochemicals can engage this established enteroendocrine framework via TAS2R signaling as operating within the canonical ileal brake.^63^

### Bitter Receptor Activation and Carbohydrate Metabolism in Humans

The multitude of data suggests that beyond the 2 primary functions of the gastrointestinal TAS2R chemoreceptors (ie, recognition of bitter toxins in the upper gut and the bitter signaling crosstalk with microbiome in the distal portions of the tract), they also contribute to the luminal content sensing in the small intestine.^64^ More specifically, because plant-based foods are the only sources of both bitter-tasting phytochemicals and carbohydrates in human diets, our earlier studies hypothesized that a particular subset of the gastrointestinal TAS2Rs gained the function to prime or modulate the body carbohydrate metabolism in anticipation of carbohydrate loads associated with bitter plant foods.^17^ This can be achieved with a direct inhibition of glucose uptake in the jejunum, where TAS2Rs, the sodium-glucose cotransporter 1, and the fructose transporter SLC2A5 (GLUT5) colocalize,^65^ or with a possible indirect effect on the low-affinity basolateral monosaccharide transporter SLC2A2 (GLUT2) that enables sugar transfer from enterocytes into the bloodstream. At the same time, TAS2Rs also colocalize in the gastrointestinal enteroendocrine cells that express and secrete GLP-1,^66^ with direct effects on insulin secretion and improved postprandial glucose responses. This hypothesis provides a possible explanation why diverse, unrelated classes of nontoxic bitter phytochemicals rapidly modulate carbohydrate metabolism while not sharing a common chemical structure or pharmacophore.^61^ In addition to GLP-1 (22% of the response), the incretin-mediated insulin response is also dependent on the glucose-dependent insulinotropic polypeptide (44% of the response) and glucose itself (33% of the response),^67^ suggesting a vast underexplored area of metabolic regulation that could be harnessed for novel dietary interventions.

Cinchona bark (*Cinchona officinalis* L.) is a bitter spice that yields alkaloid quinine found in a variety of modern drinks, including tonic water, gin cocktails, wine blends (eg, Dubonnet, Malaga Quina, Barolo Chinato), and soft drinks (eg, Irn-Bru, Paso de los Toros, Faxe Kondi). Quinine content in foods is limited to 83 mg L^–1^ in the United States and 100 mg L^–1^ in Europe.^68^ The hypoglycemic effect of quinine is known in association with the treatment of malaria^69^ and consuming gin-and-tonic cocktails.^70^ Intragastric administration of quinine at 275 and 600 mg to 15 healthy study participants decreased the glycemic response (area under the curve [AUC] = 120) to a nutrient drink by −9% to −14% (*P* = .04) without slowing gastric emptying.^71^ Similarly, both intragastric and intraduodenal administration of 600 mg quinine to 14 healthy study participants prior to a nutrient drink decreased peak postprandial blood glucose by −11% to −14% (*P* = .017).^72^ These effects were slightly more pronounced in female participants (−23.7%; *P* < .05) and were also associated with increased plasma GLP-1, CCK, C-peptide, and insulin levels.^73^

Gentian root (*Gentiana lutea* L.) is a bitter herb that contains the secoiridoid glycosides amarogentin and gentiopicrin and is widely used in bitter preparations (eg, Suze, Salers, Aveze, Amaro, Angostura) and soft drinks (eg, Moxie). The aqueous extract of the root was coated with ethylcellulose^74^ to provide 100 mg of secoiridoids to 20 healthy study participants; the result was a 30% decrease in energy intake (*P* = .04), as well as a trend for a higher GLP-1 response.^75^ A 1:1:1 mixture of gentian root, cinchona bark, and chicory root in 600 mg capsules was tested in 31 overweight individuals consuming a 40% hypocaloric diet for 90 days. Prolonged satiety occurred, accompanied by a −5.9% decrease in fasting blood glucose (*P* < .01) and −11.4% decrease in body weight (*P* < .0001).^76^

Hops flower (*Humulus lupulus* L.) is a bitter herb rich in α acids (humulone, α-lupulic acid) commonly used as a bittering agent in beer. The 100 mg and 250 mg capsules containing 51.5% α acids were given to 30 healthy, fasted study participants and resulted in a 10% reduction (*P* < .05) in the self-reported hunger scores.^77^ In another study, capsules containing 8-48 mg of isohumulones were given to 94 individuals with prediabetes daily for 4 months and resulted in a −4.6% reduction in fasting blood glucose (*P* < .05) and a −0.3% reduction in hemoglobin A1c (*P* < .01).^78^ Similar findings were observed for the model bitter substance, denatonium benzoate, after its intragastric infusion in healthy female study participants.^79^

## POLYPHENOLS, SMALL PHENOLIC ACID METABOLITES, AND BITTER RECEPTORS

The multitude of studies also point to the fact that bitter polyphenols in herbs and spices can also improve glucose tolerance by stimulating gastrointestinal hormone secretion, although many of the studies focused primarily on coffee chlorogenic acids,^80^ tea catechins,^81^ and blackcurrant anthocyanins^82^ without a direct connotation to their interactions with the gastrointestinal bitter receptors. The realization that many polyphenols and their metabolites taste bitter to a certain degree was largely obscured by the fact that this bitterness is highly variable and depends on their glycosylation status, changes in hydroxylation and methylation profiles, as well as the degree of polymerization. At some point, condensation and/or polymerization reactions in polyphenols shift the perception of bitterness toward astringency, which does not depend on direct interactions with bitter receptors but instead relies on formation of stable complexes with proteins that convey a drying or puckering sensation.

This perception has changed in the recent years as the information about interactions of different phenolic compounds with the individual TAS2Rs started to accumulate in cell culture^83^ and preclinical models^61^ and appeared in databases, such as BitterDB,^37^ dedicated to bitter ligands and the associated bitter-taste receptors. It was also used in machine learning–based prediction tools for identifying putative ligand-TAS2R interactions, such as BitterX.^84^ The current prediction algorithms routinely achieve 76%-82% accuracy, which allows for the effective modeling of large bitter-compound libraries.^85^

### Anthocyanins

A substantial number of herbs and spices are rich in anthocyanins, including blackcurrants (*Ribes nigrum* L.), roselle (*Hibiscus sabdariffa* L.), kokum (*Garcinia indica* Chois.), and dark varieties of basil (*Ocimum basilicum* L.) and perilla (*Perilla frutescens* (L.) Britton). The parental structures of anthocyanin glucosides had a high potency score for putative activation of up to 11 human TAS2Rs (Table 2). These scores diminished as the parent structures were degraded into small phenolic acids and their metabolites, accompanied by shifts in the predicted TAS2R activation profiles. The final phenolic breakdown products formed immediately prior to mineralization^86^ were predicted to virtually not be recognized by the human TAS2Rs (Table 2).

**Table 2.** Putative Interactions (% Binding Probability) of a Model Anthocyanin, Its Aglycone, and Small Phenolic Metabolites With Bitter-Taste Receptors, Calculated Using the BitterX Machine Learning–Based Model. The chromosomal localization of TAS2Rs is color coded as chromosome 5 (blue), 7 (green), and 12 (orange)

**hT2R** ^a^	1	3	4	5	7	8	9	**10** ^b^	13	**14** ^b^	16	38	39	40	41	42	**43** ^b^	**44** ^b^	45	**46** ^b^	47	48	49	50	60	**PS** ^c^
C-GA				75	73					72	65	57	76		61		67	60		62	57					319
C-R				78	75					65	61	52	69		60		60	57		54	60					304
C-G				75	67					71	65	51	74		60		66	59		61	54					309
C	62			62						73			78		58		63									95
CA	67							53		71	66		59													63
FA	68		54					60		72	70		58		54											122
DHC	67							53		69	67		53		53											87
DHF	67							57		69	69		55		56											90
COA	59		69					59		69																41
PCA	57									55																9
VA	59									57	52						53									35
PAA	68		78					76		66	64		55		58											130
HVA	64							52		71	66		54		52											86
HBA	73		53					59		77	69				56											93
BA	72		77					74		68	66				54											99
PHG										60			51													9
PG													51													2
CAT	60									59			60		57											38

a hTR2, human bitter-taste receptor family. The parent compounds were cyanidin-3-rutinoside (C-RG); cyanidin-3–(6-acetylglucoside) (C-GA); cyanidin-3-glucoside (C-G); as well as the cyanidin aglycone (C) and its small phenolic metabolites caffeic acid (CA); ferulic acid (FA); dihydrocaffeic acid (DHC); dihydroferulic acid (DHF); *p*-coumaric acid (COA); protocatechuic acid (PCA); vanillic acid (VA); phenylacetic acid (PAA); homovanillic acid (HVA); 4-hydroxybenzoic acid (HBA); phloroglucinol (PHG); pyrogallol (PG); and catechol (CAT).

b Human bitter taste receptor with broad specificity.

c The thresholded weighted potency score (PS) was calculated as sum of probabilities >50% threshold (activation strength) divided by a total number of receptors and multiplied by the number of activated receptors (activation breadth).

### Proanthocyanidins

The proanthocyanidins are another group of polyphenols abundant in herbs and spices, particular in cinnamon (*Cinnamomum cassia* (L.) J.Presl) and cocoa powder (*Theobroma cacao* L.). However, proanthocyanidins are found in these powders in an average degree of polymerization that ranges from 4 to 10, with monomers and dimers present only at the level of 5%-10% of the mixture. The thresholded weighted potency scores of these compounds suggest they do not interact with TAS2Rs at the level of trimers and above. Similar to anthocyanins, small phenolic metabolites generated from proanthocyanidin breakdown had diminished capacity to activate TAS2Rs (Table 3). This observation may explain why large doses of cinnamon are necessary to observe its effects on postprandial glycemia levels in humans (a nonsignificant −36% reduction in AUC of 0-180), and why these observations remain inconsistent among the different studies.^87^

**Table 3.** Putative Interactions (% Binding Probability) and the Potency Score of a Model Proanthocyanidin, Its Monomeric Units, and Small Phenolic Metabolites With Bitter-Taste Receptors, Calculated Using the BitterX Machine Learning–Based Model^a^. The chromosomal localization of TAS2Rs is color coded as chromosome 5 (blue), 7 (green), and 12 (orange)

**hT2R** ^b^	1	3	4	5	7	8	9	**10** ^c^	13	**14** ^c^	16	38	39	40	41	42	**43** ^c^	**44** ^c^	45	**46** ^c^	47	48	49	50	60	PS
E1^a^																										0
D1^a^												51														2
C1^a^																										0
B1				74	77					72		56	74	71	58		63	56		53	57					313
A1				73	75					69		51	67	63	51		62	61		56	58					302
CT	64			59						72	54		72		63		59									124
ECT	64			58						71	52		73		62											91
VAL	55		63					63		75			62		59		57			51						155
HAA	60									68			52		54											37
EGC	59			57						68			72		55		55									88
GCG				64	67					71			77		51		65			55						126
GA	93									98	96		87				94									94

a Large proanthocyanidin pentamers (E1), tetramers (D1) and trimers (C1) were predicted not to interact with bitter-taste receptor family members (TAS2Rs).

b hTR2, human bitter-taste receptor family. The parent proanthocyanidin dimers were B1 and A1, as well as their metabolites catechin (CT), epicatechin (ECT), 5–(3'-hydroxyphenyl)-γ-valerolactone (VAL), 3–(3'-hydroxyphenyl)-hydracrylic acid (HAA), and gallo derivatives epigallocatechin (EGC), epigallocatechin gallate (GCG), and gallic acid (GA).

c Human TAS2R with broad specificity.

### Other Phenolic Compounds

Spices and herbs also contain a particularly abundant variety of flavonols (quercetin, kaempferol, myricetin) and flavones (apigenin, luteolin), among flavonoid components. These flavonoids interact with TAS2Rs similar to anthocyanins in that the respective di- and monoglucosides are perceived as more bitter, and their predicted bitterness decreases as these structures are metabolized (Table 4).

**Table 4.** Putative Interactions (% Binding Probability) and the Potency Score of Model Flavonoids With Bitter-Taste Receptors, Calculated Using the BitterX Machine Learning–Based Model. The chromosomal localization of TAS2Rs is color coded as chromosome 5 (blue), 7 (green), and 12 (orange)

**hT2R** ^a^	1	3	4	5	7	8	9	**10** ^b^	13	**14** ^b^	16	38	39	40	41	42	**43** ^b^	**44** ^b^	45	**46** ^b^	47	48	49	50	60	PS
QR				78	72					62	52		68		57		53				53					158
QG				76	64					68	58		72		57		62	53		55						203
Q	57			66						73			76		55		62									93
API				73	71					69	58	52	70		58		62	57		58	57					301
AG	54			68	57					75	60		78		59		64			57						206
A	62			57						77			81		61		56									95

a hTR2, human bitter-taste receptor family. Parent structures were quercetin rutinoside (QR), quercetin glucoside (QG), and quercetin aglycone (Q), as well as apiin (apigenin diglycoside) (API), apigenin-7-*O*-glucoside (AG), and apigenin aglycone (A).

b Human bitter-taste receptor with broad specificity.

Lower postprandial glycemia was confirmed in clinical studies after consumption of fenugreek (*Trigonella foenum-graecum* L.),^88^ amla (*Phyllanthus emblica* L.),^89^ basil (*O. tenuiflorum* L.),^90^ and turmeric (*Curcuma longa* Linn.),^91^ among others. In combination studies with healthy volunteers who consumed 150 mg of coffee chlorogenic acid and 540 mg of green tea catechols, the acute beneficial effects on postprandial glucose (−5.4%; AUC = 0-240; *P* < .05), insulin, and incretin responses to a high-fat and high-carbohydrate cookie meals were also observed.^81^ The TAS2R-related molecular mechanisms behind these effects were also evaluated in cell culture for other glycosylated secondary metabolites, such as steviol glycosides,^92^ secoiridoid glycosides,^93^ glucosinolates,^94^ and sesquiterpene lactones.^95^ These findings indicate consuming herbs and spices that contain polyphenols capable of activating TAS2Rs may stimulate the release of incretin hormones from specialized cells in the gastrointestinal tract, trigger insulin secretion, and ultimately reduce postprandial blood glucose levels within a few hours after a meal.

## REDISCOVERING BITTER IN MODERN DIETS

Consistent with the human studies we have described, both ancestral and modern higher-quality diets are expected to be intrinsically more bitter, because they tend to include a greater diversity of plant-based foods rich in secondary metabolites. Encouraging the consumption of bitter foods or adding the desired bitterness to foods in the form of spices and herbs may help recondition taste preferences, especially in populations habituated to hyperpalatable, highly processed foods.^96^ They can be used as a means to re-expose and potentially recalibrate taste preferences in populations accustomed to highly processed foods. Over time, this could contribute to greater dietary variety, improved nutrient density, and enhanced metabolic resilience.^97^ Therefore, promoting acceptance of bitterness may be a powerful strategy to shift eating behaviors toward healthier, more sustainable diets.

**Figure 3.**
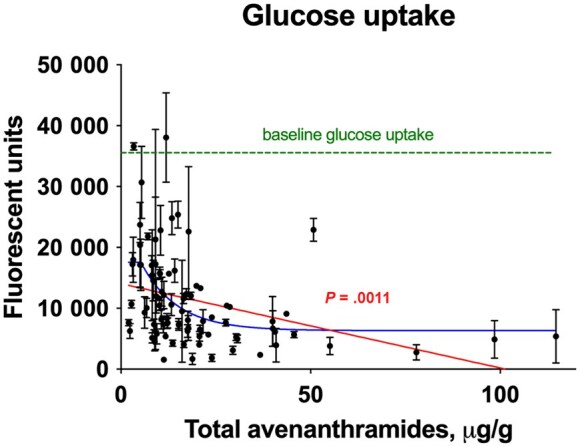
Fluorescent 2-NBDG glucose uptake in the STC-1 intestinal cell model after exposure to aqueous oat digests from the 109 and Food Research Initiative Collaborative Oat Research Enterprise phenotypic oats panel. Cells were incubated with treatments for 2 hours, presented with 2-NBDG for 30 minutes, and fluorescence was quantified at excitation/emission of 465/540 nm.

This statement also extends to modern cultivars of spices, herbs, and grains that were selectively bred for milder flavors, often at the expense of their original bitter and astringent phytochemical profiles.^27^ As a result, many of these cultivars may have reduced concentrations of bioactive compounds that contribute to metabolic health. Reintroducing or preserving the bitter traits of traditional varieties could enhance both the functional and nutritional value of these dietary staples. This is substantiated by observing the rates of glucose uptake in the intestinal cells after exposure to digests from the Agriculture and Food Research Initiative Collaborative Oat Research Enterprise oat worldwide diversity panel with different levels of bitter-tasting secondary metabolites (Figure 3).

These observations imply that the reduction of bitter phytochemicals during crop domestication may have inadvertently diminished natural glucose-regulating mechanisms and adaptive hormonal responses that optimize nutrient handling. An alternative approach to achieve similar dietary effects is incorporating select spices and herbs into foods and beverages as a practical way to reintroduce beneficial bitterness into modern diets. Unlike purified metabolites, spices and herbs deliver these phytochemicals in complex, fiber-rich or oil-based matrices^98^ that facilitate delayed release in the gastrointestinal tract, unless left to cook for a long time. Reintroducing these traditional flavors also aligns with a broader movement toward functional, health-promoting diets.

## CONCLUSION

The widespread distribution of TAS2Rs throughout the gastrointestinal tract highlights their multifunctional roles beyond taste, including site-specific effects on nutrient absorption and microbiome interactions. Activation of gastrointestinal TAS2Rs by plant-derived bitter compounds stimulates the release of key hormones such as GLP-1 and CCK, promoting better glucose regulation, insulin secretion, and appetite control. Thus, rediscovering and reintegrating bitter flavors from common spices and herbs into modern diets offer a promising strategy to improve metabolic health and dietary quality. Broadening dietary exposure to bitter phytochemicals could represent a simple yet powerful step toward more resilient and health-promoting food systems.
